# Utilising the learning in development research framework in a professional youth football club

**DOI:** 10.3389/fspor.2023.1169531

**Published:** 2023-06-08

**Authors:** Mark O'Sullivan, James Vaughan, James L. Rumbold, Keith Davids

**Affiliations:** ^1^ Department of Sport and Social Sciences, Norwegian School of Sport Sciences, Oslo, Norway; ^2^ Sport and Physical Activity Research Centre, Sheffield Hallam University, Sheffield, United Kingdom; ^3^ AIK Youth Football, Stockholm, Sweden

**Keywords:** skill learning, ecological dynamics, ethnography, talent development, affordances

## Abstract

Underpinned by an ecological dynamics rationale, the Learning in Development Research Framework (LDRF) has been suggested to introduce methodological possibilities to investigate and illuminate: (i) socio-cultural constraints within a sports organization or club, and (ii), a research gap on the need for a more contemporary framework to guide reliable ways of conducting investigations and designing practical applications. To provide a strong justification for the nature of the fieldwork and methods adopted, we present insights from a 3-year and 5-month study at a professional football club in Sweden that adapted the framework as a central feature of their Department of Methodology for player development. A phronetic iterative approach was employed to analyze the data. The findings highlight the nature of constraints acting over varied timescales, transcending contexts to manifest in other contexts (e.g., practice task designs), influencing events and experiences. This indicated a need to dampen (using probes) the influence of the pervasive organizational “control over context” approaches that were acting as “sticky” socio-cultural constraints, shaping the intentions (in session design) and attention (during practice and performance) of players and coaches. A practical implication is that the LDRF does not prescribe a universal solution to player development. Rather that it can guide how researchers, practitioners, clubs and organisations could challenge themselves to adapt strategies to design contemporary athlete development frameworks within their ecosystem.

## Introduction

It has been recognized that a sports organisation is part of a complex, multi-layered system, where the social, cultural, and historical contexts are important constraints on the development and understanding of skilled performance (1). Exemplified in the specific social, cultural, and historical traditions of a nation or region, these factors can play an important role in shaping the way coaches design practice and the way athletes engage with learning environments (2). However, sport science research has tended to undervalue socio-cultural and historical factors that influence athlete learning and development, neglecting critical features that have important implications for transferring findings to applied settings (3). For example, a path dependency of seventeenth-century scientific ideas [i.e., the Newtonian/Cartesian paradigm, see (4)] has arguably led sport science to downplay the role of environmental constraints, creating an *organismic asymmetry* (5). This biased preference for organism-centered explanatory mechanisms, focusing on the “internal mechanics” of the athlete (5), has had an influence in shaping applied research and practical interventions. This has led to a significant body of research applied to sport narrowly focusing on the individual athlete (6), resulting in practitioners and organizations holding an (ontologically) limited picture of the complexity of human learning and development in sport (7).

To counteract the previously mentioned organismic asymmetry in research and player development, ecological dynamics has emerged as a guiding theoretical framework to inform new approaches to research, athlete development and pedagogical practice in sport (8). Drawing on ecological psychology and the theory of constraints on dynamic systems (9, 10), an ecological dynamics perspective proposes that skillful behaviour emerges from the complex and dynamic interactions of an individual's continuous adaption to surrounding constraints, which change over micro- and macro-timescales (8). Here, it is implied that skill learning occurs in the midst of ongoing developmental changes within specific socio-cultural contexts (2). This perspective highlights the potential for a myriad of possible complex, unpredictable and ill-defined challenges for sport organisations when seeking to implement an athlete development framework (3).

A growing body of research encompassing sociological and ecological approaches to coaching and athlete development are highlighting a need to radically broaden our ways of knowing [e.g., (11–13)]. Indeed, more recent research has sought to highlight how environmental factors contribute to the development of expertise (see (1)) (14, 15). While these studies have proven adept at providing a descriptive account of the current context in which athletes develop, there seems to be little or no intention to initiate change or evolve practice in that context. Considering the potential for a myriad of possible complex and ill-defined challenges in the realm of talent development, there is a need for an approach that will guide reliable ways of conducting research, and designing practical applications, that reveal insights on the socio-cultural complexities and sub-system interrelatedness of athletes and environments. Until recently, no specific research framework has been proposed to help sports clubs and organizations with this endeavor. In response, O'Sullivan and colleagues (2) introduced the novel Learning in Development Research Framework (LDRF), a deeply contextualised, transdisciplinary approach to action research that is founded in ecological dynamics and the Skilled Intentionality Framework (SIF). It is our intention to advocate for and later outline, how the LDRF, refined during the first author's PhD thesis, was utilised to guide an iterative, ongoing cycle of research and action, and support the evolution of player development framework at a professional football club in Sweden.

### The learning in development research framework

Utilizing novel ways of knowing, coupled to an ecological perspective (e.g., the theory of ecological dynamics and the Skilled Intentionality Framework), the LDRF can be utilized to guide both research and practice within specific sport organisations (2, 13). In this paper we exemplify how socio-cultural practices (task designs) in a specific player development environment has adapted to, and is constrained by, social and cultural forces and how interventions, system probes, were devised to probe the system.

The LDRF focuses on ecological approaches that can illuminate the interplay between socio-cultural constraints and affordances for skill learning within a form of life. Wittgenstein's (16) notion of a *form of life*, consisting of values, beliefs and practices that continually shape how we live (17), helps us to comprehend the myriad of socio-cultural constraints that can influence an athlete's responsiveness to available opportunities for action. For example, a form of life may define dominant ways of *doing* in a society, community or organisation (17) and can be conceptualised as something that is deeply acculturated, socially accepted, and often taken for granted. Demonstrating the influence of specific socio-cultural and historical constraints in the development of expertise, current forms of life identifiable in sport could be soccer in Argentina (la nuestra) and Brazil, skiing in Northern Europe and rugby union in New Zealand. These examples provide insights into why certain performance styles are developed in certain regions and why they are valued. This notion also appears in the Skilled Intentionality Framework (SIF), a conceptual framework that, through utilizing ethnographic strategies to generate knowledge, directly couples forms of life to the relevant fields of affordances (opportunities for action) that influence skilled action [see (18)].

Within an ecological dynamics rationale, it is proposed that an individual perceives the environment in relation to its functionality, its meaningfulness detected in information for affordances (9). Affordances are properties of the individual-environment system that do not cause behavior, but constrain it (9), helping us avoid problems with defining skill development as an internal characteristic of an individual or of the environment. Foregrounding the notion of sociomaterial entanglement, the SIF highlights how affordances are not just passively situated in isolation in the materiality of immediate behavioral settings of a sports organisation (training session, competition). Rather they are deeply entwined within a more culturally encompassing, socially and historically developed constellation of practices and forms of life (18). Constitutive sociomaterial entanglement is the ontological notion used to explain the active, dynamic and transdisciplinary reality of the environments in which we live and develop. It proposes that the ways which we live (forms of life), the practices we partake in (sports training methods), the affordances we perceive (invitations for action in these contexts) and the skills we develop (e.g., passing, dribbling) are constitutive relations and aspects of a holistic system that continuously form each other (2, 18). So, while a form of life can influence the way sports organisations implement their athlete development frameworks, the SIF helps illustrate the extent to which socio-cultural-historical constraints in a form of life (e.g., a football club) shape the intentions of players, soliciting some affordances over others and directing skill learning in development (2).

**Figure 1:**
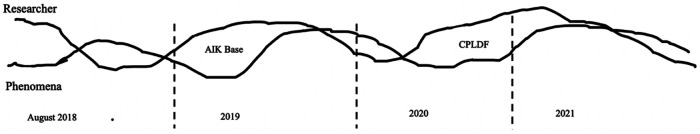
The LDRF foregrounds research that is “undergone” longitudinally through attentive and responsive participation. How the researcher comes to know the landscape, and how they learn to correspond (probes), is through dwelling. The graphic highlights how the LDRF was utilized, through an ongoing ethnographic inquiry punctuated by probes (AIK Base and Contemporary Player Learning in Development Framework) delivered at the micro-level of on-pitch coaching pedagogy. Inhabiting a place in-among the coming-into-being of the phenomena (as it evolves and persists), encourages a perceptual attunement to its ebbs and flows, and what the phenomena have to share directly with the researcher. Graphic is adapted from Woods et al., (2022).

To elaborate on these ideas, it is useful to consider data from a 3- year and 5-month study in a professional football club in Sweden that has adapted the LDRF (see Figure 1 for graphic outline) as a central feature of their Department of Methodology (DoM), to support the evolution of a player development framework. As outlined by O'Sullivan and colleagues (2), we first endeavour to understand the “practical situations” within which behaviour emerges within this organisation. To achieve this, the SIF, foregrounding aspects of qualitative inquiry (i.e., ethnographic) are introduced to unpack and enrich our understanding of the relations between coaches behaviours, the socio-cultural and historical context, and players' intentions/interactions within a relevant field of affordances. As the intention with the LDRF is to initiate change and/or evolve practice, the impact of being immersed in a local setting, utilizing the SIF, is complemented by an action cycle that aims to implement its findings. Considering that macro-level socio-cultural constraints evolve over the years and can be difficult to directly influence, we illustrate how DoM devised interventions to probe the system (17) at the micro-level of on-pitch coaching pedagogy. For instance, respectively amplifying or dampening helpful and unhelpful aspects of form of life that are acting as socio-cultural constraints on coaches' intentions (in session design) and players' attention (during learner interactions in the sessions themselves). A simple example of this could be damping language that amplifies ideas and narratives associated with socio-cultural constraints e.g., referring to under nine football players as elite (see (21).

Highlighting the highly iterative and integrated nature of the LDRF, as findings are being implemented through system probes, in tandem, the next research cycle (utilizing SIF) seeks to capture the evolving sociomaterial environment as it persists and changes, connecting the research back into the next action cycle. More directly, while ethnography supports long-term immersion in a local setting, it can have limited impact (19). When complemented by action research, ethnographic research is more “likely to be useful and usable by those working on the ground … and to address the identified gaps between research and the ability to implement its findings” (23). This combination of ethnographic strategies and action research can offer a deeply contextualized and continuous analysis and assessment of a form of life in a particular ecological niche, even while findings are being implemented [for a more detailed discussion see (2)].

We contend that emphasizing the enrichment of a reciprocal and functional relationship between athletes and environments forming complex, interconnected systems (24) (e.g., ecological dynamics), can provide a valuable theoretical underpinning for action research (25). In turn, action research can provide a valuable methodological approach to the critical and practical study and evolution of complex systems in contexts like sport, work and education (25). It is this opportunity of analyzing the phenomenon in greater depth each time (highly interconnected research-action cycles), illuminating the influence of a form of life at the microscale of development (i.e., how players engage with affordances for skilled behavior) and support interventions to probe the system (e.g., player development environment) that characterises the Learning in Development Research Framework (LDRF) in practice.

The Athlete Talent Development Environment (ATDE: (1)) was used as a framework for data collection and organisation. Acknowledging Feddersen and colleagues’ (2021) (26) work on the limitations of the ATDE, we extended its use, emphasising the ecological level of analysis by embracing a Gibsonian perspective (9), with particular emphasis on (17) relational view of affordances in the SIF. Here, affordances are not just passively situated in isolation in the materiality of immediate behavioral settings of a sports organisation (training session, competition), but are entwined in a particular way of life (18).

Rietveld and Kiverstein (17), extended the more traditional *action-scaled* view on affordances, suggesting that affordances are not simply action opportunities offered by the environment but are dependent on the “abilities available in a particular ecological niche”. To illustrate how each sporting context is contained within its own form of life, which may amplify or dampen player engagement with some affordances, it is worth considering Winner's (27) rich narrative of Dutch football. As a mirror expression of its culture, architecture, landscape, history, politics, geometry and dance, the idea of “total football” was built on a new theory of flexible space, creating space where there was not any before^1^ (28). The theory of affordances embedded in forms of life can provide a powerful rationale to help practitioners consider the socio-cultural constraints in specific environments, which may shape expectations and beliefs on coach and athlete behavior, performance, development and learning.

A central aim of the LDRF is to shift the focus of athlete development research away from just the individual athletes and towards understanding behavior at the level of interactions between a performer and their performance environment, both continuously shaping each other (29). The SIF provides the philosophical foundations for this shift as described by the de ontology of constitutive sociomaterial entanglement (18). This world- view aims to demonstrate the extent to which the ways we live (forms of life), sociocultural practices we participate in (e.g., football), opportunities for action (affordances) and the skills we develop exist as, and exhibit, a constitutive relation where practices and affordances do not admit of a prioritization (2, 18, 30). For example, referring to Winner (27), it can be suggested that the Dutch playing style of “total football” evolved, within a specific socio-cultural context as players' perceptual systems and effectivities developed in, interaction with an intention to create a diverse range of passing and dribbling opportunities/affordances to exploit space.

The SIF can make a profound contribution by providing a perspective on the extent to which athlete development environments are sociomaterial and constitutively entangled within broader macro contexts and structures (1, 6). By connecting social and cultural aspects of life with the skill development of athletes, the SIF helps to demonstrate the resonance between a form of life and the relevant field of affordances that stand out in their training sessions. This resonance has been explained as the value-directedness of player-environment intentionality [see (30)]. An ecological account of these dynamics is reflected in this paper, where for example, we outline how expectations that embody a cultural inheritance of player compliance towards prescribed coaching methods can shape a value-directedness toward affordances that can partially realize that value [(26); for an example see (2)]. Overtime players that are exposed to these practices may develop unskilled intentionality (coordinate with only a narrow range of affordances (30).

The SIF significantly contributes to the way one views an ATDE by outlining the extent to which the intentionality of any organism-environment system is an interdependent and constitutive relationship (18). In other words:

Intentionality characterizes the system, not just biological organisms within the system. Thus, intentionality in the sense of value-directedness characterizes environmental structures [i.e., a form of life/ATDE] and processes [i.e., sports training methods] as much as it does the organisms [football players] who shape and are shaped [e.g., skill development] by those structures and processes. This implies that values are necessary constraints on both the constitution and the selection of affordances [(26) text in brackets added)]

We must aim to comprehend, and attend to, the contextual complexities of our situations and co-create practices that amplify and dampen helpful and unhelpful aspects of our form of life. Aligning with North and colleagues (32) warning against the uncritical application of good practice ideas from other successful countries, this approach recognizes that there can be no “copy and paste”.

## Materials and methods

### Background and context

Allmänna Idrottsklubben (AIK) football club in Stockholm, Sweden provided a rich and unique socio-cultural and historical backdrop for this study. AIK youth football engages around 1,700 players between 5 and 19 years. In April 2017, after an in-depth, rigorous analysis and review of its operations at child-youth level, the club disbanded its early talent selection policy and set about investigating possibilities to build a player development framework guided by three strategic goals: (i) to support the well-being of the child; (ii) to follow supporting documents from the United Nations Convention on the Rights of the Child and Swedish Sports Confederation and (iii), secure the promotion of more youth players to participate in the under 16, under 17 and under 19 years teams and in the clubs senior teams.

In January 2018, the newly formed AIK Department of Methodology (DoM), consisting of professional coaches and sports scientists, was introduced in the club's yearly planning document. To support the club in its endeavor, the LDRF was adopted by the DoM to investigate the current athlete development environment and to inform present and future possibilities of evolving practice and player development.

### Access and role clarification

The study was given institutional ethics approval^2^ and all interviewees provided informed consent prior to participation. The first author gained access due to his dual role of practitioner-researcher and member of the DoM, as part of their terms of employment at the club. The potential for role conflict throughout the research process due to this dual role was recognized. For example, it could be perceived as a position of authority over various staff members, having an influence on the data collection process (33). However, the value the club placed on the importance of researchers and coaches collaborating in practice was consistently reiterated by the head of youth development at the twice-yearly coach and parent education meetings.

### Research design and procedure

To illuminate the interplay between socio-cultural constraints and affordances for skill learning, we present a 3-year and 5 months inquiry (see Figure 1) to illustrate how a professional football club adopted the LDRF as a feature of their DoM. Ethnography, as a central feature of inquiry in the LDRF, can broaden the scope of ecological psychology by providing methods that can theme the patterned practices of a form of life in an organization and provide insights into athlete-environment intentionality (13, 18). Ethnography is founded on the collection and documentary of data (audio, video, field notes, interviews) predominantly relating to what Gibson (34) theoretically refers to as secondhand *knowledge about* the environment. Comparatively, *knowledge of* the environment is reflective of embodied-embedded knowledge developed by, and exemplified in, activities behaviour that enhance the coupling between perception and action (34).

This epistemological distinction is apparent in the differences between *knowing about* the environment through indirect perception of information (9) that has been produced and documented by another person (typical of coach education courses). This form of knowledge embodies the manifestation of an external relational but “generalised” environment. In contrast, *knowing of* (9) the landscape's invitations to act directly engages with perception and action (i.e., attuning to information) in a specific performance environment (35). Araújo et al. (29) proposed that gains in direct perception (e.g., gaining knowledge “of” one's environment) may be mediated through communication *about* the environment. Information collection via ethnographic endeavour can be used to create themes that can uncover broad socio-cultural constraints that influence the value- directedness of player-environment intentionality which, in turn, frames the perception-action couplings for affordance utilization (13).

To understand how players respond to affordances offered by the material aspects of the environment and by other people (in practice and competition), it is important that we understand the practical situation in which behavior occurs (18). Embedded here is the appreciation that training sessions, competitive games do not take place in a socio-cultural vacuum but are deeply entangled within meaningful contexts of a broader societal form of life (37). The ethnography (utilizing the SIF) employed in this study allows for a rich exploration of the extent to which social and cultural patterns of life are embodied in the way football is played and skills developed (18, 30).

As the intention with the LDRF is to initiate change and evolve practice in a specific athlete development environment, the impact of being immersed in a local setting was complemented by an action cycle that aimed to implement its findings. Here, with the support of a DoM interventions were devised to probe the system (17), to amplify or dampen socio-cultural constraints shaping the form of life. Ethnography provided insights into the form of life, and as subsequent probes were implemented, helped to capture real-world changes in practice and connect the research back into the development of a player development framework.

To summarize, in order to engage with the sociocultural complexities and sub-system interrelatedness of athletes and environments, we have outlined the relationship between socio-cultural constraints, player-environment intentionality and fields of affordances. We have argued for a combination of ethnographic strategies and action research to offer a deeply contextualized and continuous analysis and assessment of a form of life in a particular ecological niche, even while findings are being implemented. We will now briefly highlight the ethnographic strategies of inquiry that help us link a zoomed- out view on the form of life to a zoomed-in perspective on concrete situations (micro systems of practice). We will then proceed to illustrate how the LDRF informed present and future possibilities of evolving practice and player development at AIK youth football.

### Historical contextual analysis

It has been argued that to better understand athlete development in and through sport, culture and context matter most (29, 38). A contextual historical analysis (available as extra material) has been proposed as a productive approach for investigating the socio-cultural contexts in which phenomena historically unfold (12). This lens provided insight into the overarching ecological context (macrosystem) that conveys the information, ideology, and values that influence organizational structures (i.e., roles, responsibilities, tasks) and events in the embedded microsystems (i.e., coaching sessions) where athlete development takes place (38).

Information was retrieved from various documents and media such as books, coach education material and articles (both printed and electronic) sourced from the Swedish Football Association (SvFF), Swedish Sports Confederation (SSC), AIK football club and various newspaper articles relating to child youth sports in Sweden and the development of Swedish society, which the first author translated from Swedish to English. The most important sources were The Nordic Sports Forum archive^3^ on Swedish sports, sport policy and sports studies, the Swedish Sports Confederations document bank^4^, the Swedish Football Association's (SvFF) coach education material, the Center for Sports Science (CIF) archive^5^ and various national media archives (e.g., Dagens Nyheter^6^ archive from 1864 to 2022). These sources contributed towards illuminating some unique social, cultural, and historical constraints that informed observation and interview methods and what data should be collected in the field.

### Observation

Unobtrusive observation provided the opportunity to observe behaviours and actions, sociocultural practices and events (39). Participant observation does not require a specified group of participants (33), and initially offered the opportunity to follow the participants across several contexts (training, match day, meetings, informal office conversations). These types of public observations pose no threat to neither the observer or the observed (33) and people are not identifiable within the data (41).

During the first action cycle (probe), the first author adopted the role of observant participation (35) to support on-field education, enabling deeper insights into the functioning relationships, rules and peculiarities of the place and people, all of which are fundamental to ethnographic research (43). Field notes included text, audio and video recordings, reviewed and categorised into a detailed log of field notes by the first author within 36 h of events occuring (44). This promptness helped to inform the development of the data and how the first authors' emotions, experiences and assumptions might have influenced the creation of knowledge (46). For example, often on the train back from training the first author would begin to edit the filmed training session, while making some additional notes to be worked on the next day. Video clips from practice sessions were edited and shared with coaches on a regular basis to initiate further discussion and reflection.

### Interviews

Informal conversations occurred spontaneously in the context of participant observation (46). For example, this included, speaking with coaches before and after practice sessions, at club educational events or during an impromptu “fika”^7^ at the office. A feature of these unexpected situations and chance encounters is that they can contain less asymmetrical power relations than more structured interviews (47). Participant consent was sought from key club colleagues and leaders, who are represented (with pseudonyms) but not identifiable within the data (41). After purposeful sampling of participants, ten semi-structured interviews were carried out to probe for richer information. The selection criteria were that the participants must be an active coach, must work actively in the setting and have attended the Swedish Football Association coach education courses. Six coaches held a UEFA A coaching license, one held a UEFA B, and three coaches had a level one (C-Diploma) qualification. The interview guide was inspired by Henriksen (1) and the first author's knowledge of youth football in Sweden. A 3-part guide for semi-structured interviews was formulated. The first section sought to elucidate the participants' experience of playing football as a child (e.g., “What did a typical training session look like when you were a youth footballer?”). The second section aimed to explore participants' entry into the coaching world and their experience of coach education (e.g., “were the type of practices promoted on these coach education courses evident in AIK?”). The third section explored participants' experiences since AIK took the decision to restructure its youth football in 2017. As a fluent speaker, the first author carried out the interviews in Swedish, later transcribing them into English. Interview lengths varied from 30 min to 45 min.

Throughout the study the first author returned to the interviewees to gather more data, where informal interviews and conversations, filming and assisting during training sessions, helped me to achieve more depth and comprehensiveness, increasing contextual depth in the research (48, 49). Swain and Spire (47) highlighted how the role of informal conversations in qualitative research is contested but also relatively neglected. However, considering the deeply embedded nature of their dual role of practitioner-researcher, the first author regarded informal conversations as opportunities to hear people “tell it as it is” in an everyday context (50), potentially providing “context” and “authenticity” that can enrich the data (47). Indeed, Hammersley and Atkinson (50) further argued that these interactions or conversations are still a type of interview, in the informal sense. Informal conversations can be seen as a useful way to establish a rapport, gain trust, reduce the imbalance between the researcher and participant and get closer to the reality of individuals' experiences, perceptions and beliefs (51, 52).

### Data analysis

A distinct feature of many social science inquiries has been the sequential nature of data collection, analysis and writing up of studies (45). However, in this study a phronetic iterative (a cycle that repeats) approach was utilised, alternating between emergent emic (e.g., “ a nerve”) readings of the data and an etic (e.g., coach centered coaching) use of existing models and theories, was used to analyse the findings (45). Analysis alternated back and forth between: (1) data generation, (2) scrutinising emergent findings from the data, and (3), consulting existing theoretical and conceptual frameworks underpinning this case (i.e., ecological dynamics) (45). The ethnographic process supported the commencement (during research-action cycles) of data analysis to begin with data generation (54), helping us to identify promising directions of research (45). Initial analysis began with a formal process of interpretation, a descriptive “primary cycle coding” or “open coding” (45), where descriptive and basic codes were developed. Examples included, “focus” and “everything in order/ordning och reda”. During data collection, the Department of Methodology, which included the second author, regularly met in various constellations, as critical friends, to offer different perspectives, reflexively acknowledge multiple “truths” (55) and discuss emerging interpretations. This helped in determining how the initial primary cycle codes might be developed in the process of “secondary cycling”. In the secondary coding cycle, the first author began to interpret, organize and synthesize codes (45). This move towards more “focused” coded themes required interpretation and theoretical considerations. In particular, the first and second authors' understanding of theory and literature provided a foundation for interpreting and building theoretical explanations, as well as informing new lines of inquiry (45). For example, we can endeavour to devise an umbrella code, a larger hierarchical code called “coach centered pedagogy” that can encompass smaller primary codes like “focus” and “everything in order”. Fundamentally, this iterative approach to data generation and interpretation informed the refinement and development of system probes and helped capture the evolving sociomaterial environment as it persists and changes, connecting the research back into the next action cycle (probes).

**Figure 2:**
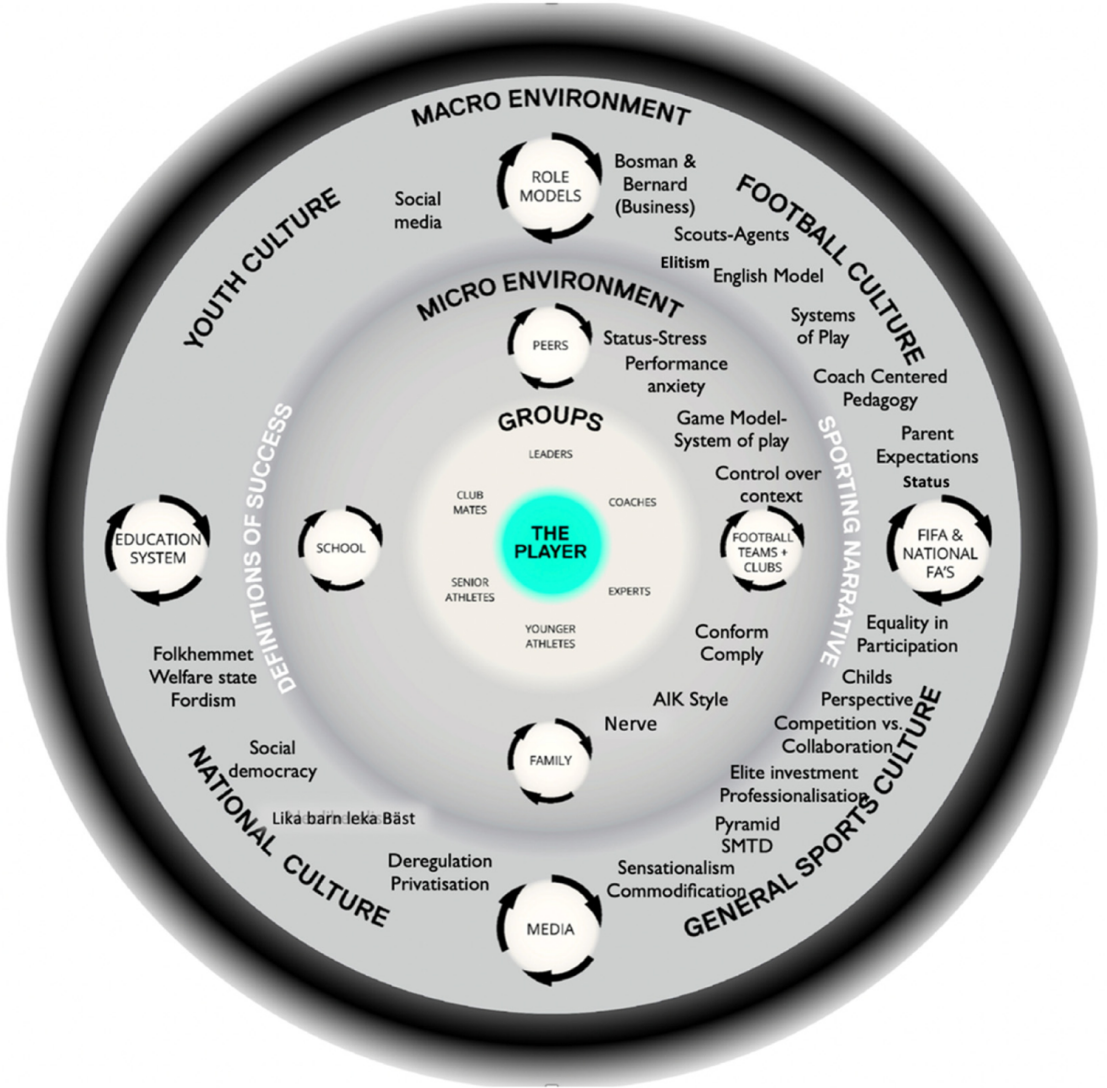
AIK ATDE used to organize and locate the data within a broader ecological context. Themes are embedded in relation to the environment/context (macro or micro) in which the data emerged and cohered.

We turned to Henriksen's (1) adaption of Bronfenbrenner's bioecological model (56), the Athlete Talent Development Environment (ATDE) to assist with the interpretation of themes. The AIK ATDE (Figure 2) was used as a formative model for data collection, organisation and presentation of themes, that signifies their context (e.g., macro or micro) of origin or significance [see (13) for details].

### Qualitative rigor

Considering the potential subjectivities, the first author brought to the research process and the writing of the paper, they took steps to be reflexive and sincere in making sense of the data and drawing plausible conclusions (57). The first author's expertise in youth sport and coach education, both nationally and internationally, brought unique insights into the underlying meaning of the sociocultural factors on skill development. For example, the first author gained their UEFA B coaching license in 2011 and UEFA A coaching license in 2013, both from the Swedish Football Association (SvFF). From March 2015 until June 2018, they worked as a head coach educator in the Stockholm district, delivering the first two levels of the new SvFF education courses (launched 2014).

This dual role implied prolonged embedded engagement, dwelling in the context of the phenomena, which can promote accurate and truthful depiction of the participants' lived experience (58). The third author's qualitative research experience and the DoM acting as critical friends, contributed to the reflexive process (59). Indeed, a specific feature of this study was the first author shifting from participant observer to observant participant (during on field education). It could be argued that the first author had succumbed to “going native”, an occupational hazard with ethnography (60). However, Moeran (35) argued that this shift enables the researcher to gain access to information and knowledge that is otherwise available only to insiders, and that the very richness of the data collected and interpreted from observant participation will always overcome this disadvantage. This view aligns with the transdisciplinary approach central to the LDRF, encouraging the researcher to engage directly with the phenomenon, opening unique lines of inquiry.

## Results and discussion

We now illustrate how the LDRF was used to illuminate the relations between coaches behaviors, the socio-cultural and historical context, and players intentions/interactions within relevant fields of affordances. Expanding on, and refining the work of Vaughan et al. (13) we focus on the pedagogy of practice in the microenvironment and zoom out to consider the path dependent influence of these socio-cultural constraints on practice. This complimentary perspective presents a new set of data gathered within AIK's ATDE (see Figure 2), enriching our interpretations and guiding practical interventions conceived as system probes (17). Interventions conceived as system probes aim to skillfully amplify and/or dampen helpful and unhelpful aspects of forms of life, often these aspects can be characterized as socio-cultural constraints within the framework of Ecological Dynamics (2, 11, 13).

The meta theme “control over context” is presented in the microenvironment but represents a coherence of data, illustrating the cascading influence of (value-directed) themes throughout cultural sub- systems (national culture, sports culture, football culture). This helped create a context that led to the emergence of “context-dependent” constraints (61) (e.g., types of task designs, development pathways, expectations), shaping the value-directedness experienced by players.

We will now exemplify this idea, presenting data highlighting a value directedness relating to the club's microenvironment as well as the broader football culture in Stockholm. Value-directedness is an important aspect of player-environment intentionality (intentions) that shapes intention and guides attention toward certain affordances (2).

### Status and performance anxiety

I am with Vincent (academy coach) watching the boys 2008 academy training. Parents are anxiously peaking over the closed off 7 a side pitch, while standing on benches in the adjacent children's playground. Something happens and one parent looks up to the night sky, while the parent next to him stares at him and opens his arms, then drops his head and shakes it. The 2008s are the last group to go through the early academy selection before AIK raised the academy age. It was in the local newspaper Mitt i Solna, that AIK head of youth development Leif Karlsson, explained the reasons behind this decision was to “dampen the emphasis on child-youth football being about children being assessed^8^ “[Document analysis: Mitt i Solna article: AIK höjer åldern för start i akademilagen, June 2017 (62). Translated from Swedish]. Indeed, I recall an article I read on the Swedish football associations (SvFF) homepage^9^. It referred to how “youth football in Stockholm is the most stressful and unhealthy in the country” [Document analysis: Fogis, September 2017 (63). Translated from Swedish].

With the end of the season approaching in two months, and an imminent selection/ de-selection process approaching, the intensity is quite high. The last part of the session seemed to be a game of constant transitions. The early part of the session was a lot of repetitive predetermined passing patterns followed by 1v1's with no consequence (once the attacker lost the ball the 1v1 was over).

Vincent: This is more like a tennis match

Mark: The coach seems to be willing them on, tempo! tempo!

Vincent: This, I guess is what performance anxiety looks like coming up to the end of the academy season. They are competing against each other, even when in the same team, instead of collaborating to make each other better.

(Field note: Informal conversation, September 21st, 2018).

These results reinforce the findings of Vaughan and colleagues (13) by demonstrating ways in which performance anxiety can emerge from a value-directedness towards interpersonal competition, amplifying opportunities and behaviours that maintain or protect social status. However, these types of observed behaviours also evidence how, shaped by specific socio-cultural constraints, the structure of development pathways and implemented pedagogies went “hand in glove”. This close relationship between pedagogy and player development pathways was further highlighted in the coach interviews, as exemplified by Coach A:

You see when I began [2011], there was a little nerve that influenced pedagogy. You were forced to have results even at 9, 10 years. A lot of decisions were pre-decided – very clear predetermined patterns which we also practiced very hard in training

The description of a little “nerve” that influenced pedagogy, is indicative of an anxiety and expectation (e.g., results) that cascaded through organizations and structures, amplifying ideas associated with early elite investment and selection de-selection [Standard Model of Talent Development/SMTD, see (64)]. The SMTD, is characterized by early selection into exclusive training programs that often promote hyper-specialization and result in eventual deselection (55, 76). Indeed, this model was a central feature of AIK youth football between 2009 and 2017 where children were “selected for the youth academy one year at a time” [Document analysis: AIK Verksamhetsplan, 2011 (66). Translated from Swedish].

Coaches maintained their status through results and adopted deterministic methods in training and games to control future outcomes and limit unpredictability. The same “nerve” also emerged in parent behaviour, where ideas of linear causality in task designs (limit unpredictability) and “nivåindelning” (the best playing with the best), appealed to an emerging parenting style, a form of “monitoring” to ensure exclusivity with their child not having to encounter too much resistance and problems in their development (67). This is captured in the theme ‘lika barn leka bäst’ (alike plays best with alike), a commonly used cultural phrase that could convey the idea that children of the same ability should only play and train together. “Lika barn leka bäst” is categorized within a single theme in connection to the national culture but transcends across three themes in the general sports culture (SMTD, Elite investment and Competition v collaboration) and two themes in the football culture (Elitism, Parent expectations/status) manifesting as a form of exclusivity to maintain control, promoting conformity and compliance in the microsystems of practice. When projected onto youth football this phrase, part of the vernacular in and around youth football, could amplify a value on early selection practices associated with the SMTD that were fast becoming a continual pervasive practice in Swedish youth sport (55).

### Lika barn leka bäst

Mats a parent of one of the young players (9 years old) approaches me. At a club parent meeting, he let his opinion known about AIK removing the early selection model, saying that the club will “lose the best players to other clubs if we don”t select early” (field note 06-11-2018). He was very clear about the need for the best to train with the best. Arne, who coaches this group, had mentioned to me that he had a few parents that wanted “nivåindelning” (splitting the kids into cemented ability levels). His feeling was that “this is about winning games for their own child”. (Field note: Informal conversation, February 13th, 2019). Once again, Mats emphasises the point he made about the need to select the best with the best.

Mats: We call this “saft och bulle” (children's soft drink and buns) training. It's better to split the group into those that are more motivated and those who just want to play. (Field note: Informal conversation, February 14th, 2019).

Interviews further highlighted parental expectations relating to how young players’ learning, and development “should look” in practice. As highlighted by Coach H.

Through a lot of years in football. My feeling is that parents who don't have proper insight into development, they like the look of organisation. They like the look of control, which is easy to get because you can put them in a line and do a passing drill and for someone who doesn't have insight, it can look very, very good. You also get an effect from that very, very quickly and the players can look quite good, quite quickly for doing stuff like that.

Coupling observations to the explanations of coach A and coach H, we can appreciate how a “nerve”, symbolic of the broader intentionality of a form of life, has impinged on coach intentions when designing and delivering training sessions. This broader intentionality is further illuminated via document analysis relating to a congruence of entangled themes (“status”, “SMTD” and “elite investment, professionalisation, adultification”) that sit in tension with themes relating to the democratic values of Swedish sport (“equality in participation” and “child's perspective”), in the Aftonbladet newspaper it was reported that:

There is currently a professionalisation of Swedish youth football with foreign big teams, child stars used as advertising planks and agents who shadow football pitches with money in their eyes. The ideals that Swedish youth football movement is based on has been split in two. On one side are international big clubs, elitist academies and money-hungry agents who have turned sports into a market and children into consumers and products. There is an evolving culture of status among parents that have a “high performing child”.

[Document analysis: Aftonbladet article: Blivit status att ha ett presterande barn, November 24, 2019 (68). Translated from Swedish]

It can be suggested that these tendencies, a system intentionality/ value-directedness (towards early professionalization) that characterize environmental structures (organizational structures within an ATDE) and processes (training sessions), mirror those from broader macro levels beyond Stockholm and Sweden. For instance, the “Bernard case” [see (69)] in 2010, a follow up to the Bosman ruling, had helped to frame youth football as an economic activity, encouraging the training of young “talented” players as a form of “processing” human capital investment. This arguably contributed to the legitimisation of a player development system underpinned by the notion of “early elite investment” in young “talented” Swedish footballers**,** where young children are grouped by ability into an “elite” group (lika barn leka bäst). Indeed, it was sports psychologist Johan Fallby that described a “distorted system” in Stockholm youth football, one driven by agents and money and “a strong culture based on anecdotal evidence that early selection works”.

[Document analysis: Expressen article: Den allvarsamma leken—vem är det som inte får vara med? 31st October 2018 (70). Translated from Swedish].

We are moved to propose that this emergent distorted system in Stockholm youth football was shaping and amplifying ideas and expectations as to what young players learning in development in youth football should look like in practice. Coaches adopted deterministic approaches under the assumption that inviting players to interact with a narrow range of affordances could provide the mirage of “control” and improve learning and performance, which in the footballing form of life, was associated with results. One way to limit unpredictability regarding results was to have an early selection of the best children with the best. This value-directedness toward control over context, through limiting unpredictability aligned with a deeply rooted path- dependent coach education form of life and promoted a pedagogy that had deep innate aversion to uncertainty and ambiguity. The main problem is that this reductionist approach deprived children of opportunities for skill adaptation, a fundamental basis for motor learning.

### Coach centered pedagogy—a path dependent coach education form of life

Practices prioritized in Swedish Football Association (SvFF) coach education until 2014 (coach education was reviewed) were underpinned by a culturally dominant planning paradigm (e.g., specific themes, predetermined coaching points), predetermined passing patterns and the notion of “optimal” technique, enhancing player compliance by using explicit corrective feedback. These practices highlighted a cultural-historical inheritance that had a cascading influence on the type of practices promoted and appreciated in youth football. This approach can be traced back to the 1970s, when the pedagogical legitimacy of SvFF's “Swedish model” (based on the West German model) was being questioned by the successful sporting results and the seemingly more professional nature projected by the “English model” (introduced to Sweden by professional coaches Bob Houghton and Roy Hodgson). The English model promoted a “teacher-centered” pedagogy, where the coach had the overall picture of how the game should be organized and the players needed to comply and internalize the systematized knowledge about football performance that the coach promoted (60).

Exemplified in the intricacies of the “technical register” (coaching folder and video archive of 31 films^10^, see footnote), these ideas dominated coach education until 2014. Coach H elaborates:

It [technique register] was almost like a workbook on every type of isolated technique which should be used in football. We [the coaches on the course] were doing sessions on tackling [theme]. And we were standing in lines. The first in line ran towards the ball and tackled, kicked the ball, and we were told [by coach educators] how to do that. So yeah, it was, yeah, wasn't good, a lot of instructions, a lot about telling players what to do.

The interviews highlighted the role of the “technique register” as a “gold-standard textbook” of ideal movements, promoting a reliance on external agency (i.e., high levels of instruction and feedback) in coach education, and a reductionist and mechanistic attitude towards practice and performance. This perspective contributed to the amplification of a coaching culture that attempted to control future outcomes, shaping beliefs and expectations, while influencing the formation of practices at the club, as summarized here by Coach G: “The technique register, and its micromanagement, was absolutely seen in AIK, in the everyday practices.”

Highlighting a system capture (doing things the way we always have done them), coaches were following how they have been coached or complying with the approaches of more “experienced” coaches. Here Coach C reflects on his early years at the club as an assistant to more “experienced” coaches.

When I started coaching, at the beginning, I didn't reflect on this [coaching like how I was coached]. I did isolated training. My first year, 2016, was with XXXX. There were some drills that were A to B to C passing, and you must do this technique. Not very representative. A lot of go, go, go and this is how we do it, this is a good drill as it goes quickly. It looked good, organised, it gave a little boost, but I never felt it gave much really.

The idea of “it looked good, organised”, aligned with cultural expectations of what practice should look like. A consequence of this continuous reinforcement loop was the culturally resilient belief, that greater stability and consistency in match performance is related to practising repeatable movements or patterns, Coach B refers to how this belief was evident in the more “theme-driven” game-based designs that was limiting player engagement with affordances.

Yes, even now when they say to players that we must work with switching the play [theme], players just pass the ball from side to side all the time because this is what the coach thinks that they must do and the players' understanding is limited by the idea that they must switch the play, but they will do it so that it will look right for the coach.

One of the main aims of the new SvFF coach education courses, introduced in 2014, was to support coaches understanding how “Task designs that have the game as the starting point contribute to conditions for increased learning” (72). However, strong socio-cultural influences were arguably contributing to a system inertia. A dissonance between coach educators' socially and culturally constructed beliefs and SvFF's intention to contemporize coach education was evident. Even the more game-based task designs promoted on these new courses were arguably prolonging the shelf life of traditional inherited beliefs about how skill is understood and acquired. Coach C elaborated on his experience attending the UEFA B course in 2018.

We had an “overlap” themed session. When we were finished, we were asked by the coach educators if we were happy with what we saw. We said relatively happy! Then we got criticized for a lack of successful overlaps. The coach educator assumed that the success of an overlap was when the overlapping player received the ball. The idea of a successful overlap is not about receiving the ball but also about distracting the defenders, pull them out of position and create other opportunities i.e., a gap to pass or dribble through as the defense is moved out of position by the overlap. We were also criticized for not clearly mentioning the theme of the session in the introduction.

**Figure 3:**
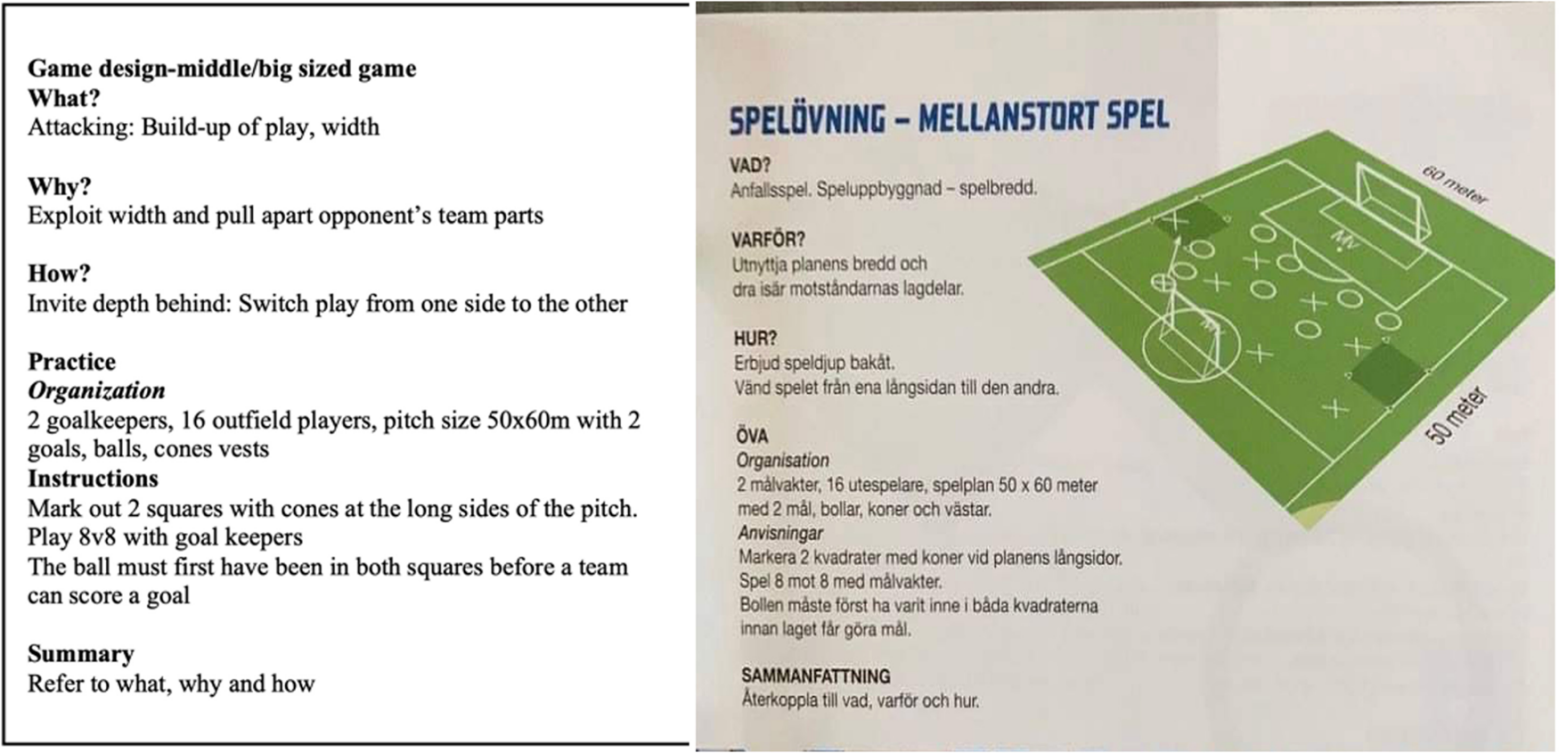
Svff UEFA B session design (SvFF UEFA B Coach education, p. 43).

Intentions to challenge coach educators' socially and culturally constructed beliefs were not helped by the promotion of artificial task constraints in some task designs to limit unpredictability (control over context). This is captured in the coach educator's assumption in the definitive statement “that the success of an overlap was when the overlapping player received the ball.” Another example taken from the same UEFA B course (see Figure 3), highlights the use of the rule that the ball must be played from one side of the pitch to the opposite side *before* a team can score a goal. The key problem with this type of “universal” instruction is that, with attackers being invited (by the pre-determined rule) to attack wide areas, defenders may co-adapt their movement behaviours to deal with this rule and change their positions to defend the wide areas at the expense of central areas. Despite best intentions, task designs on coach education courses continued to embody a “control over context” path dependency, limiting player exploration opportunities and contributing to the maintenance of a traditional, hierarchical model, the position of the (controlling) sports pedagogue at the heart of the learning process.

### First probe: AIK base (underpinning practice within a theoretical framework)

**Figure 4:**
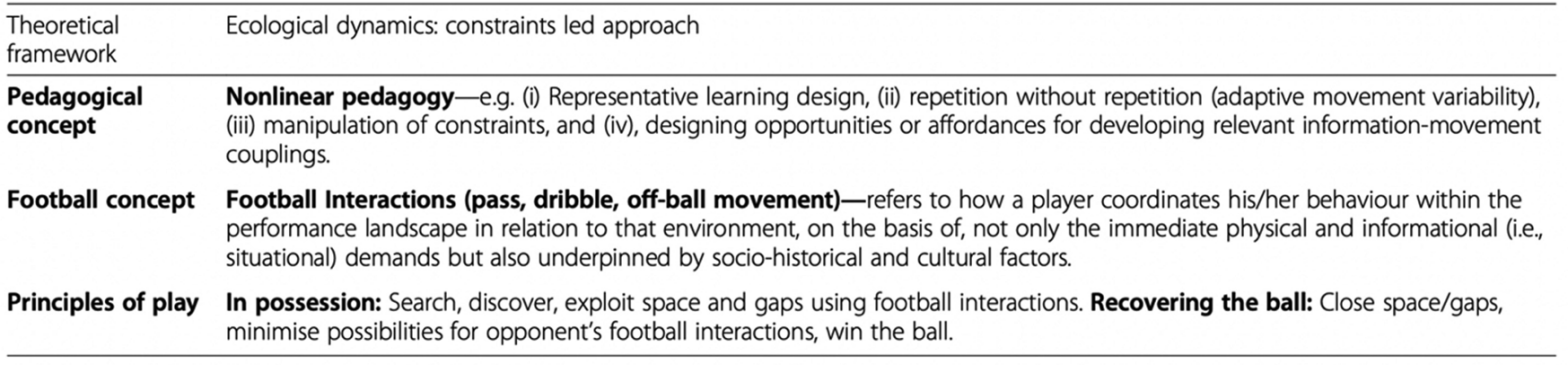
The theoretical framework underpinning AIK base (taken from Woods et al., 2020) (75).

Findings from the initial research phase indicated a need to dampen the influence of the “control over context” approaches that were acting as socio-cultural constraints, shaping the intentions (in session design) and attention (during practice and performance) of players and coaches. Considering that macro-level socio-cultural constraints evolve over the years and can be challenging to influence, the DoM focused on the micro-level of on-pitch coaching pedagogy, and in particular practice task designs. To form a coherent foundation for the club's practice design and education programs the “AIK Base” framework (Figure 4) was created to encourage the coordination of shared principles and language. Beginning in October 2018 (see Supplementary Material), DoM began providing in-house coach education with “on field” support throughout 2019, where the integration of the research to support the development of a player development framework was explored.

Rothwell et al. (11) pointed out that player development pathways could benefit greatly from underpinning their practice within a theoretical framework of the learning process, to mitigate dominating influence of socio-cultural-historical constraints. Grounded in the theoretical framework of ecological dynamics, coaches were encouraged to adopt methodologies and principles of a constraints-led approach (CLA), informing a nonlinear pedagogy in practice (73). The ecological notion of *Football Interactions* was introduced to help shift the narrative away from implementing predetermined “optimal” prescribed actions (e.g., football culture, such as “English top-down model”), towards developing a more adaptive performer. Football Interactions acknowledge that everything that happens on the football pitch is an interaction and these interactions take place in a broader ecology of interactions, beyond the playing area, that shape development within overlapping forms of life. Further, football was defined as a dynamic team sport, in which players routinely flowed between attacking and defensive phases of play. This dynamic offensive and defensive flux, underpinned by the ecological dynamics framework and informed by a modified three-stage learning model, search and exploration; discovery and stabilization; exploitation (see 74), informed “principles of play” at AIK youth football.

### The “sticky” nature of socially and culturally constructed values, beliefs and attitudes

As initial interventions (AIK Base) to probe the system were being implemented, in tandem, the next research cycle (utilizing SIF) sought to capture the evolving sociomaterial environment as it persisted and changed. Due to the inherent, ecological complexity of a form of life, a probe may or may not initiate the change intended, meaning one cannot impose a specific course of action, only probe, sense, and then respond (17), implying that a probe may or may not initiate the change intended. One of the aims with AIK Base was to inform how coaches can design in affordances to support skilled intentionality (coordinate with a broad range of affordances simultaneously). However, path dependencies evident in socio-cultural practices that were anchored to a dominant “coaching” form of life, meant that encultured approaches remained at times challenging to change and were very “sticky”. The notion of sticky refers to an ideological inertia, shielding traditionally inherited beliefs about how skill is understood and “acquired” (76).

This “stickiness” was revealed in the “over- constraining” of practice tasks through the application of the game model concept at the club. A game model has been described in the literature as an overarching strategic approach and tactical principles of play, considered of fundamental importance for team organisation to enhance player functionality in specific sub-phases of play (77). The theme “game model” is categorized in the microenvironment, nested between the themes “control over context” and “compliance” (see Figure 2), illustrating a value directedness of themes throughout cultural sub- systems e.g., football culture (“English top-down model”, “systems of play”, “coach -centered pedagogy”) and the general sports culture (“professionalisation”), towards limiting unpredictability.

We earlier highlighted a form of game model, where young players were drilled to recall pre-determined passing patterns to be later regurgitated in competitive games [see (2) for more details]. The following section relates to data highlighting how a game model was being implemented in the academy, amplifying player compliance, while aligning with a contribution of practices associated with what was culturally understood as professionalism.

Meeting with Ragnar (head of development for the boys academy). He showed me some session designs that the coaches have logged in to XPS. In general, the designs looked good but many of the sessions had detailed pre-determined coaching points. I wondered “how much insight these presentations of training sessions give into what actually happened in training Are the coaching points a box ticking exercise?” (Field note: 19 January 2020).

Mark: Coaches are clearly spending a lot of time on planning, editing clips and administration work.

Ragnar: What is it we have discussed before? The illusion of professionalism?

Mark: It would be interesting if coaches added in observation and reflections after the session- what happened?

Ragnar: Certification points means lots of administration for the coaches and money [for some it's their salary] is connected to certification. Some of the administration might be of benefit but there is too much.

(Field note: informal conversation, 19 January 2020)

There is a major tendency with coaches to discuss “What” to coach (e.g., tactics, game model), what equipment do we need (footballs, cones). “How” to coach is rarely discussed. I guess that the “How” is harder to administrate! I recall what the Head of academy recently said, “the illusion of professionalism” (Field note: 19 January 2020).

I attend a coach education event organised by Ragnar. The older academy teams (U16, U17 and U19) are presenting their annual plan and how they are implementing a game model in training and in games. Adam, one of the coaches, goes through a few videos of some of the groups most recent games and training.

Adam: We have players filling all the channels [according to the Game Model] when we are in possession, but we still have problems securing control of the ball. We also have problems moving over in defense, we are slow to act.

Bart the U19 head coach presents his training based on the Swedish Football Associations work plan model. I am intrigued to know why he uses this planning model.

Bart: We are not the best at preventing the opponent's build-up of play in this club.

We are in the right position so the players think that this will take care of itself.

(Field note: informal conversation, February 3, 2020)

On a fika break I engage in conversation with Bart

Mark: I see that you are using the SvFF planning model

Bart: Yes, it works well for me

Mark: What do you mean by that?

Bart: Well, it gives structure to the training by having a clear plan to follow. It's about sticking with the plan.

(Field note: informal conversation, February 3, 2020)

The following day I bump into Coach B, now the new u16 coach, on his way out to training. We discuss the previous days presentations and discussions.

Coach B: Players are in the right position [according to the Game Model], so some coaches and players think that everything will take care of itself.

Mark: The coaches seem to emphaise organization a lot, especially in their planning.

Coach B: There is more talk about organization of the players on the pitch, organisation of planning, organisation of administration, than actual football. This is how it has been at the academy for the last few years.

(Field note: informal conversation, February 4, 2020)

During the interviews Coach B had highlighted his concerns on how a form of game model was limiting players but was also establishing a basis for evaluating players. In other words, those players that could conform to a coach-imposed game model had a better chance of surviving the selection and deselection process in the academy

There can still be better solutions outside our game model. This also limits our players and our way of evaluating players. based on the game model. It can absolutely be a part but on the other side limit players.

I was talking about this (with Coach C) and how our game model is quite “fuzzy”, where players themselves get to make many of their own decisions from the game from what they discover in the game which I believe will give them more when they are older.

As previous coach interactions with the form of life placed a value on the utilization of deterministic approaches to limit unpredictability, these inherent tendencies shaped how coaches implemented a game model. This was having an over-constraining influence on player-environment interactions. These insights align with a void in the literature identified by Ribeiro and colleagues (77), where there is a lack of understanding of how a game model may impact as potent constraints in shaping self-organisation tendencies within sports teams. While players may be taking up the “correct” position in each situation (knowledge about), in accordance with the game model, this does not necessarily imply that they will be able to self-regulate their co-positioning (based on *knowledge of* the performance context) in relation to the continuous local interactions that change at a faster timescale. Foregrounding *knowledge about* the performance context, coaches were prioritizing the operational procedures of coaching, rather than to its actual practice, arguably leading to a *system capture* e.g., doing things the way we have always done them (78).

### Second probe: towards a contemporary player learning in development framework

The second research cycle highlighted the need to dampen tendencies to prioritise knowledge about (emphasizing global to local tendencies in the team) the environment, while amplifying task designs and coach behaviours that promote the development of players knowledge of the environment (emphasizing local to global tendencies between players). Both levels of interaction are intrinsically connected, and their co-existence can lead to successful team performance (77) if global to local tendencies are manifest in “educating intentions” of players. However, through the use **of** a game model, coaches were assuming that they could improve affordances by making them more prominent to players so that they only respond to specifically designed ones. This form of “control over context”/limiting unpredictability, was depriving players of decision making and problem-solving opportunities, minimizing the coupling of perception and action in order to self-regulate behaviour. For example, players being in the right position according to the game model but experiencing problems with their positioning in relation to the fast-changing information. The rigid nature of how a game model was being implemented disregarded the interaction of individual, environmental and task constraints that shape skilled intentions from moment to moment.

Designing tasks which are more *neutral* in terms of outcomes (inviting selection from many possible actions) could better simulate the constraints of the competitive performance environment. In the continued and iterative effort to build on the key ideas of AIK Base, the Contemporary Player Learning in Development Framework [cf. (80)] was proposed to encourage coaches towards more “neutral” task designs, supportive of athlete functionality. A specific feature of this framework is the Foundations for Task Design Model (80), supported by the relational concept of shaping skilled intentions (30), that aims to support the designing of tasks underpinned by neutral affordances (79). The DoM proposed that these ideas could act as a counterweight to find the balance between providing structure or stability (e.g., game models or plans) that constrain players decision making and variation or instability through generating uncertainty and unpredictability representative of competition, in task designs.

**Figure 5:**
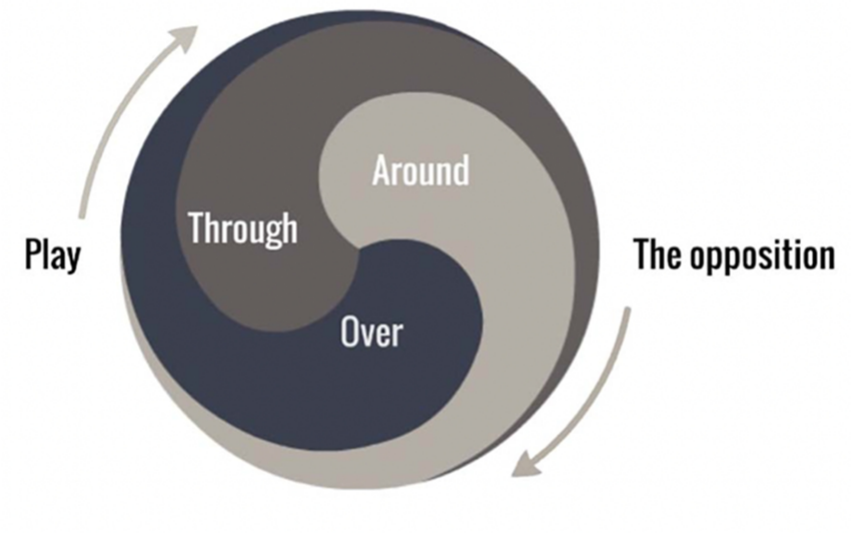
An illustration of the constitutive and nested relation of skilled intentions to play through, around and over the opposition in football [from (30)].

**Figure 6:**
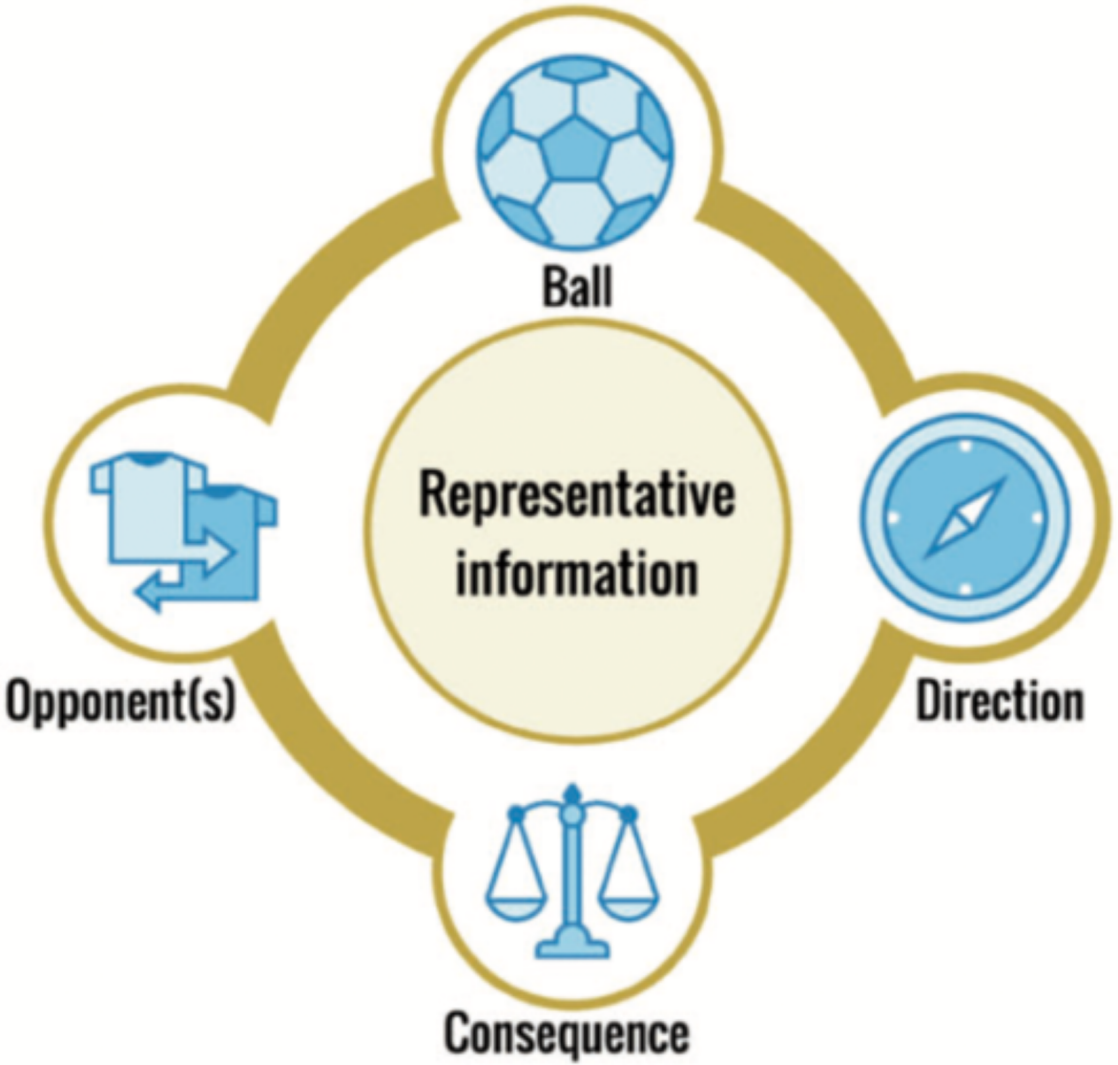
Foundations for task design model. Note. Ball-opponent(s)- direction are key aspects of task design that shape learners’ intentions and attention. The idea of consequence (e.g., if we lose the ball and do not win it back, the opponents may score), highlights the continuity and co-adaption of attack and defence. Key information in task design is representative of the game (from Sullivan et al., 2021, (80)).

Compared to methods relying on verbal instructions of abstract concepts privileging secondhand knowledge about the football environment (76), shaping skilled intentions (Figure 5) to guide attention toward dynamic properties of a football environment may be considered as an improvement in the how and why of coaching practice, as it emerges from a more accurate ontology of football and skill learning in development (30). Players display skilled intentionality through skillful responsiveness to multiple nesting and nested affordances simultaneously (30). When designing practice tasks, this approach implies that learning skills should not be looked at as a process of repeating and rehearsing a solution, but more about repeating the process of finding the solution from many that immediately emerge in the neutral affordance landscape. To further encourage this approach, the Foundations for Task Design Model (Figure 6), based on the key principles of nonlinear pedagogy, was proposed to guide the designs of football specific tasks.

## Concluding remarks

In this paper, we provided an example of how the Learning in Development Research Framework (LDRF) can guide research and action (probes) to capture real-world changes in practice and support the transfer of findings in an applied setting. We illuminated multiple and intertwined unique constraints across interacting systems that transcended disciplinary boundaries and shaped the ecological niche at AIK youth football. For example, forms of life recognisable within player development structures, coaching practice, and behaviours, amplified a value directedness that was rerouting Swedish youth football towards a form of premature professionalism/early elite investment.

The LDRF offers the opportunity for the adoption of an ecological scale of behaviour analysis, with the aim to understand human action in the very contexts (and cultures) that behaviour occurs. The potential it offers practitioners to become more aware of the extent to which unique socio-cultural constraints continuously shapes their work, can support sports organisations to enhance awareness of, and adapt to, these important environmental constraints. In this way, the LDRF offers the potential for the development of a research culture through knowledge mobilization—the act of moving research into the hands of research users. This approach implies that there are no “copy and paste” templates in performance development methodologies. Athlete development frameworks should evolve in interaction with the sociocultural and historical context in which individuals are embedded.

### Strengths and limitations

The results from this study are simply not a generalization across youth football clubs, even in Stockholm. Indeed, socio-cultural and historical constraints that influence player development may even vary from neighboring club to club (mesosystem constraints), due to the unique nature of how different forms of life can interact in a variety of socio-cultural contexts. Indeed, different organizations and clubs will present different opportunities and challenges regarding the implementation of the LDRF, particularly in relation to resources (financial barriers, access to qualified staff) and stakeholder patience (e.g., the growth of knowledge that helps practitioners to understand and identify the socio-cultural constraints, is likely to take time).

We recommend that future research should look for innovative ways to implement and refine the LDRF model across a broad range of sports and sporting contexts at various levels. For example, advances in modern technologies offer great potential towards rethinking and extending how we can carry out such deeply contextualized research as foregrounded by the LDRF. Using tablets, phones or laptops, individuals can become ethnographers in their own community, in their own time. For example, the Wayfinder platform^11^ promotes the notion of communities as ethnographers, inviting individuals in a community to contribute stories specific to their own context. Here, members of a community get to determine what is significant and interpret their own material. The potential this form of distributed ethnography offers to consider different types of knowledge and data, may illuminate insights in how to challenge inherent inertias often related to the “stickiness” of dominant socio-cultural-historical constraints. For example, in the context of this study future research could investigate what happens when coach pedagogy aims to promote skilled intentions (skilled intentionality) in football. Can coaches adopt a methodology that prioritises knowledge-of and foregrounds learning in performance, what would this look like in practice, subject to the changing contexts, situations and constraints of the real world in which coaching, learning and performance take place.

The LDRF provides the possibility to enrich the potential for co-creation (researchers and practitioners) of practice, supporting the development of a research culture through knowledge mobilization—the act of moving research into the hands of research users. Aligning with the notion that the LDRF does not prescribe a universal solution, we hope that these suggestions can further guide how researchers, practitioners, clubs and organizations could challenge themselves to adapt strategies to design contemporary athlete development frameworks within their ecosystem.

## Data Availability

The raw data supporting the conclusions of this article will be made available by the authors, without undue reservation.

## Footnotes

^1^ https://www.thoughtco.com/polders-and-dikes-of-the-netherlands-1435535

^2^Sheffield Hallam University ethical approval (Converis ID: ER5584185)

^3^ https://idrottsforum.org/merom/svidropol/

^4^ https://www.rf.se/bidragochstod/Dokumentbank

^5^ https://centrumforidrottsforskning.se/kunskap-om-idrott/rapporter

^6^ https://arkivet.dn.se/

^7^ https://www.bbc.com/reel/video/p0bmzygz/the-swedish-tradition-that-can-make-you-happier-at-work

^8^The traditional democratic values of Swedish sport, namely equality in participation and a child’s perspective.

^9^ https://fogis.se/arkiv/startsida/2017/09/fritt-spelrum/

^10^ https://www.youtube.com/watch?v=U9UGWHQX9-c

^11^ https://wayfinders.network/blog/concept-feature-overview/
