# Supplementary material for: Utilising the learning in development research framework in a professional youth football club
Source: Front Sports Act Living. 2023 Jun 8;5:1169531. doi: 10.3389/fspor.2023.1169531 (PMC10285168; doi:10.3389/fspor.2023.1169531)
Supplement: Supplementary file 1 [file Datasheet1.pdf]

Presentation of match preparation and training session designs at the academy utilizing the Foundations for Task Design Model and the Shaping Skilled Intentions Model.

Coaches were asked to present their recent training and match preparation sessions with the purpose of nurturing both global-to-local and local-to-global synergies. More directly, how they used their *knowledge about* the game to design practice tasks that guided player search and exploration, to develop players *knowledge of* the game. From the coach's perspective, the aim of practice task design was to help players embody skilled intentions that reveal nested affordances from moment to moment as they coordinate their movements both on and off the ball. From the players perspective, the aim of the practice task was to play through, around and over the opposition in possession and prevent the opposition from doing the same when recovering the ball.

Here, Ragnar describes the *knowledge about* the opposition (obtained from scouting) in relation to the team's match preparation:

> They wanted us to play outside [around], so it was easy for us to play on the wings, but it was crowded between them [through]. So, our priority was to threaten/provoke through and over and then it would be easier to exploit through, around or over. This was to put them out of balance so that they couldn't dictate that we only play the wings.
> . (Field note: October 10th, 2021)

U19

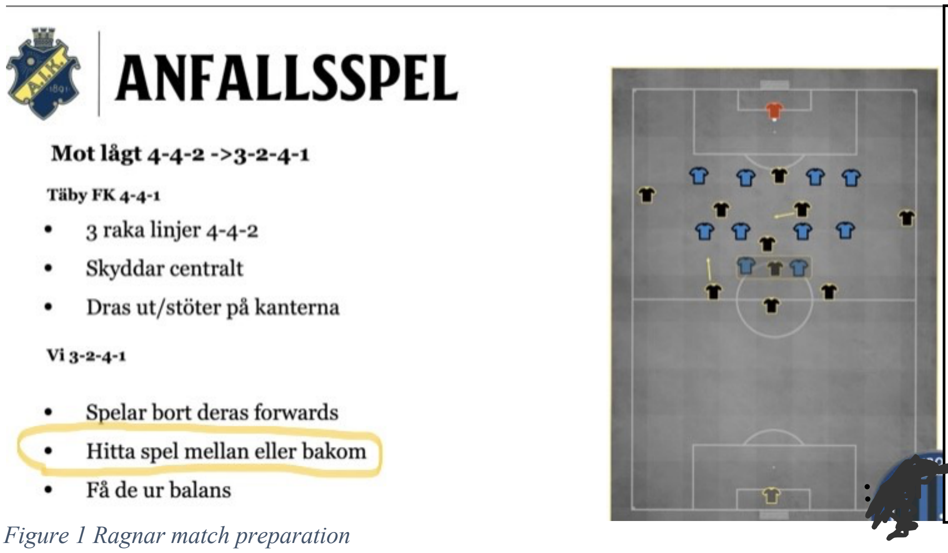

Against low 4-4-2 – 3-2-4-1
Them 4-4-2
- 3 straight lines
- Protect centre
- Pull wide/support om the sides

Us 3-2-4-1
- Play away their forwards
- Play through and over
- Get them out of balance

Figure 1 Ragnar match preparation

Coach A presented his ideas for attacking structure (against a low defensive line) that he is working together on with the players in training and match preparation. The focus was on designing tasks where players could create and exploit an imbalance in the opponents defensive organization by guiding their attention to opportunities that the opponents invite for playing through, round or over.

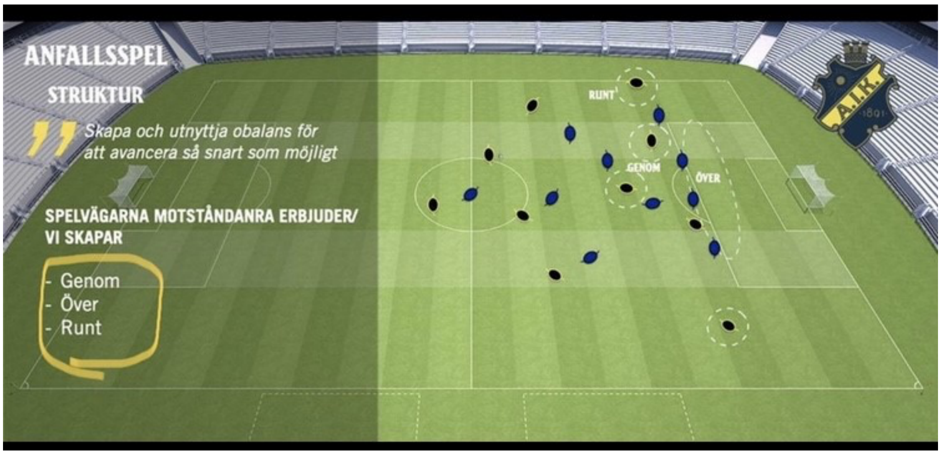

Attacking play
Structure
Create and exploit imbalance to advance as soon as possible

Opportunities opponents invite/we create
- Through
- Over
- Around

Coach C presented different sized games from 4v4 to 7v7. He provided the following rationale for his choice:

> As this is the youngest age group in the academy, I really want to place a focus on local interactions (local to global) as opposed to a general game model (global to local)
> (Field note: Informal conversation, October 10th, 2021)

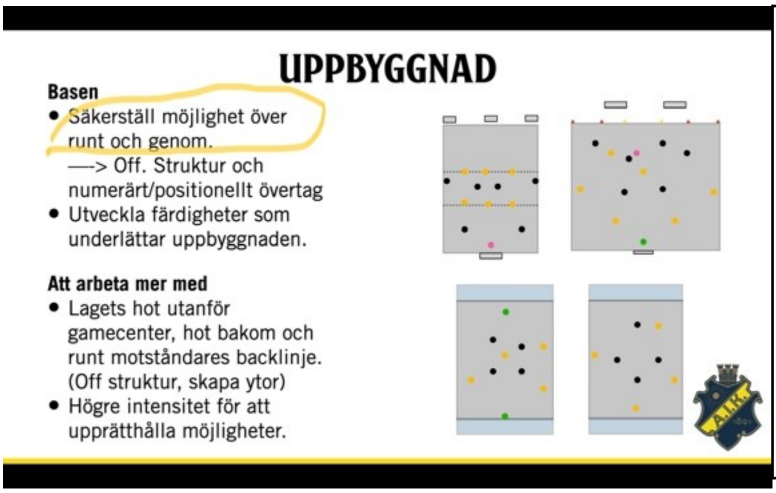

Build up play
The Base
- Ensure possibilities to play through, round and over
- Attacking structure and numerative/positional overload
- Develop skills that make build up easier

To work on
- How the team threatens away from the centre, threaten behind and around opponents backline. (offensive structure, create space)
- Increase intensity in order to establish possibilities
