# Supplementary material for: Utilising the learning in development research framework in a professional youth football club
Source: Front Sports Act Living. 2023 Jun 8;5:1169531. doi: 10.3389/fspor.2023.1169531 (PMC10285168; doi:10.3389/fspor.2023.1169531)
Supplement: Supplementary file 2 [file Datasheet2.pdf]

Educational evening presented by the Department of Methodology (All youth coaches in attendance)
(Field note: 24-10- 2018)

AIK DoM

DoM 1	Coach/Development manager
DoM 2	Coach/Development manager
DoM 3	Decision maker/Administrator
DoM 4	Researcher/Sports psychologist
DoM 5	Decision maker/Administrator
Me	Researcher/Sports pedagogue

The occasion was opened by the head of youth development, who gave a description of what is happening in AIK. It was once again clarified that by delaying the selection of players, AIK takes the initiative to be at the forefront of child youth football, with a high ambition and belief that we will to a greater extent provide an even better and even more equal and inclusive player education. Elaborating on this introduction DoM presented the quotation: “If the captain's highest purpose was to safeguard the ship, then he would keep it in the harbor DoM presented a brief description of the development work being carried out by the DoM. For example, the ideas around pedagogy and task design that we had been working together with the coaches at the AIK extra training sessions these last 2 weeks. It was further clarified by DoM 4 that DoM is about bringing people together from different disciplines and take a transdisciplinary approach.

A question was then asked to all the coaches in attendance -If we understand the development of youth football at AIK as an interaction between people and its environment, the coach and others in and around AIK, we must ask the question - what type of environment, what type of leadership and what type of pedagogy can best promote learning, creativity and motivation?

Within the following discussion came a question from Coach C about how best to help young players to become more skillful, to develop “game intelligence”?

DoM 5: The individual’s interaction with its environment is how we frame development. This means, of course, that development does not take place on a single occasion or in a linear fashion. We can only reflect on and reason about what in a given situation best contributes to genuine learning. What is worth mentioning in the context is that it is the actual process of seeking relevant information and then acting on it which is where learning is most likely to occur.

This provided a segway in to introducing the tentatively titled ‘the Base’ (later AIK Base), a concept that placed an emphasis on task designs that foreground local interactions as opposed to global concepts. The ambition is to start implementing these ideas in 2019. Videos from recent AIK extra training sessions offered to the 8-12 age group were shared. Being

proficient in the base is a starting point for over time the best way, to be able to perform a game idea/model in the 11 a side.

The Base
It is as simple as either the team has the ball, or the team has not got the ball.
We are either in possession or recovering the ball.

I introduced the term football interactions. The main purpose for this was to support moving the narrative away from a form of life based on (football) actions (as highlighted in coach education) towards a form of life based on (football) interactions. The term makes it clear that playing football is something we do together, and that it requires an understanding of the individual and their background. Everything a player does on the pitch is linked to information about teammates, opponents structure/positioning, the ball. This information then guides players solution choices.

We went a little deeper into what we have tentatively termed 'The Base'
Intercept a pass, close space, drive/dribble the ball, pass, press, cover, cover ball in 1v1, pass between two opponents or dribble between them, open space for a teammate, if you have time and space receive on the far foot, player in front (target) to pass to and behind (point guard). These are very good examples of interaction in the 'base'. Identify information that, in player-environment interactions, results in effective behaviors to use to in different ways, together, move forward to finally create goal chances or preferably make goals.

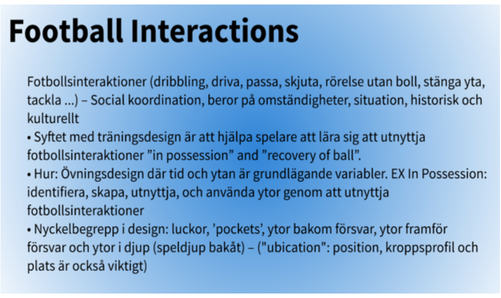

Football Interactions

Football interactions (dribbling, drive, pass, shoot, off ball movement, close space, tackle)- Social coordination, depends on circumstances, situation, history, culture

- The aim of practice designs is to help players to learn to exploit football interactions when "in possession" and " recovering the ball"-
- How: Task designs where time and space are foundational variables. Ex, in possession: identify, create, use and exploit space utilising football interactions
- Key concepts in design: Gaps, space, pockets, space behind defenders space in front of defenders and space in depth to play back.- ("Ubication"- position body profile and positioning).

Figure 1 Original slide explaining the concept of Football Interaction presented to AIK youth coaches on 24-10-2019

To emphaise the influence of socio-cultural-historical factors on the utilisation of football interactions Coach A referred to a player (16 years old) that he coached as a 9-year-old when he was part of the AIK early selection model. The coach, feeling under pressure to win games based a lot of his sessions on repeating predetermined passing patterns that players regurgitated in the game. It was successful and they won most of their games. However, the players rarely got to make decisions in the game as they had to comply with the coaches "game model". Coach C says that he can see now that many of these players don't really play information in the game, often missing opportunities to exploit gaps and spaces to penetrate the opposition.

I then presented (while referring to the question asked by Coach C) principles of non-linear pedagogy. There is no right way to perform football interactions, which means that a trainer's task is to design an environment where players can explore different ways of navigating and managing a given situation for themselves. The training should therefore, as a starting point, be information-driven, i.e., that information in task designs should simulate information in the game. This place demands on training design where relevant information sources such as teammates, opponents, ball are available and must be present in order to be detected.

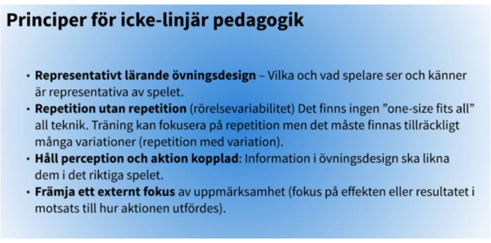

Principles of nonlinear pedagogy

- **Representative learning design**- What players see and feel is representative of the game
- **Repetition without repetition** (movement variability). There is no "one size fits all" technique. Training can focus on repetition but with variation (repetition with variation)
- **Keep perception and action coupled**- Information in the task design should simulate the real game
- **Prioritise an external focus** of attention (focus on the effect or result instead of how the action was carried out)

Figure 2 Original slide explaining the main principles of nonlinear pedagogy presented to AIK youth coaches on 24-10-2019

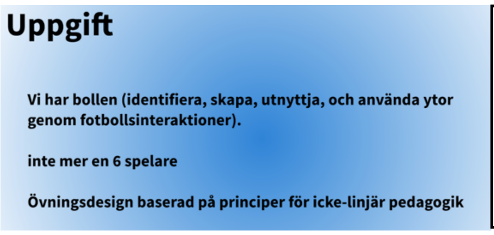

Task

n possession (identify, create, exploit space/gaps utilizing football interactions)

No more than 6 players

Task design based on the principles of nonlinear pedagogy

Figure 3 Original slide showing the task assigned to coaches on 24-10-2018

Coaches were invited to design a session on the idea of exploiting gaps/space (6 players) Coaches are invited to participate in creating and sharing a training. design bank that we want to build during the year.
