# Supplementary material for: Utilising the learning in development research framework in a professional youth football club
Source: Front Sports Act Living. 2023 Jun 8;5:1169531. doi: 10.3389/fspor.2023.1169531 (PMC10285168; doi:10.3389/fspor.2023.1169531)
Supplement: Supplementary file 3 [file Datasheet3.pdf]

Coach comments on SvFF UEFA B task design (28 October, 2021) when being examined through the lens of the Foundations for Task Design Model and Shaping Skilled Intentions.

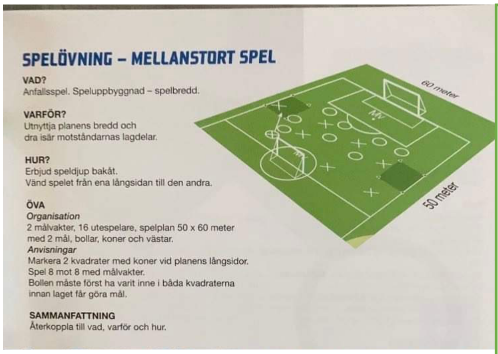

SPELÖVNING – MELLANSTORT SPEL

VAD?
Anfallsspel. Speluppbyggnad – spelbredd.

VARFÖR?
Utnyttja planens bredd och dra isär motståndarnas lagdelar.

HUR?
Erbjud speldjup bakåt.
Vänd spelet från ena långsidan till den andra.

ÖVA
Organisation
2 målvakter, 16 utespelare, spelplan 50 x 60 meter med 2 mål, bollar, koner och västar.
Anvisningar
Markera 2 kvadrater med koner vid planens långsidor.
Spel 8 mot 8 med målvakter.
Bollen måste först ha varit inne i båda kvadraterna innan laget får göra mål.

SAMMANFATTNING
Återkoppla till vad, varför och hur.

Game design-middle/big sized game
What?
Attacking: Build-up of play, width

Why?
Exploit width and pull apart opponent's team parts

How?
Invite depth behind: Switch play from one side to the other

Practice
Organization
2 goalkeepers, 16 outfield players, pitch size 50x60m with 2 goals, balls, cones vests
Instructions
Mark out 2 squares with cones at the long sides of the pitch.
Play 8v8 with goal keepers
The ball must first have been in both squares before a team can score a goal

Summary
Refer to what, why and how

Coach B

I think the focus ends up wrong here.
Already under "Why?" becomes the first problem for me, the focus is on utilizing the breadth of the pitch. In other words, a focus on the size of the pitch instead of the goal, gaps between opponents, etc. For example, to move the opponent to one side (attract), to create conditions for attacking the other (where the space is).
Number two is, as I said, that the instructions/rule force the players to switch the play for the sake of switching the play. Not based on relevant information, i.e., use space on the opposite side (Due to no possibility to play through or around on the same side). Should we not advance if we have the opportunity?
What could have been done instead is to create a super clear intention in defending teams not to allow play to go through or around. This means that they will have to move a lot to close the possibilities to play forward. That is, the opportunity opens for the attacking team to quickly move the ball to the opposite side where the space is (to score).

Coach C

When I sit down and design an exercise, it is important for me to try to make the challenge representative of the nature of the game. That the players are presented with challenges that do not conflict with the basic intentions of the game football and other invasion games, i.e., you want to advance towards goals and get past the opponents when you have the ball possession and vice versa when the team tries to recapture the ball. The challenge of the exercise is to reinforce and promote certain aspects of this, rather than working towards it.

When I see the example from SvFF's Coach Training UEFA B, it is clear that the rule works against this, the nature of the game. Situations that offer you to advance towards the goal and get past opponents are not allowed as you need to get to both squares first. This leads the players, in order to solve the task, to search for information that is not representative of the game of football. You attack in a non-representative way and defend in a non-representative way. With this, the learning you want takes place on the wrong grounds. For me, it is a clear artifact of a traditional coaching paradigm where one separates complex phenomena that should not be separated, a reductionist perspective.

There is a difference between an action and an interaction. In SvFF's exercise, the act of switching the play is separated from representative information, it appears from this perspective that it is enough that the action of "switching the play" takes place for you to practice it / learn. It is enough that the ball goes from one side to the other.
I argue that there is a difference between the act of switching the play, just playing the ball from one side to the other, and the interaction of switching the play, the team takes advantage of an opportunity to play around the opponents.(meaning/value)
One separates the game from its context while the other keeps it connected to the environment and is something that occurs naturally. Since SvFF's example separates the game from representative information, you can just as easily stand without an opponent and "switch the play", if that is the purpose.
The challenges that players are exposed to in training must be representative of the game. Players must carry the same intentions as they do in a match to search and act on the same sources of information. Then we ensure that learning takes place on good foundations.
